# Universal vaccine against respiratory syncytial virus A and B subtypes

**DOI:** 10.1371/journal.pone.0175384

**Published:** 2017-04-06

**Authors:** Jeong-Yoon Lee, Jun Chang

**Affiliations:** Graduate School of Pharmaceutical Sciences, Ewha Womans University, Seoul, Republic of Korea; University of Iowa, UNITED STATES

## Abstract

Respiratory syncytial virus (RSV) is a major cause of acute lower respiratory tract infection in infants, young children, and the elderly. Two subtypes of RSV, A and B, circulate alternately at 1-2-year intervals during epidemics. The attachment glycoprotein (G protein) of RSV is one of the major targets for immune responses. In this study, we generated a recombinant fusion protein, GcfAB, which consists of the central regions (a.a. residues 131–230) of the G proteins of both RSV A (A2 strain) and B (B1 strain) subtypes, and investigated immunogenicity, protective efficacy, and immunopathology. We immunized mice with GcfAB plus cholera toxin as a mucosal adjuvant via intranasal (IN) or sublingual (SL) routes. The IN group showed higher levels of RSV G-specific antibody responses, including serum IgG and mucosal IgA, compared with the SL group. On the contrary, more vigorous RSV G-specific CD4^+^ T-cell responses were elicited in the SL group than in the IN group after RSV-A but not RSV-B viral challenge. Furthermore, the SL group showed more pulmonary eosinophil recruitment and body weight loss than did the IN group after RSV-A challenge. Both IN and SL immunization with GcfAB provided potential protection against both subtypes of infections. Together, these results suggest that vaccination with GcfAB via an IN route could be a universal vaccine regimen preventing both RSV A and B infections.

## Introduction

Respiratory syncytial virus (RSV) is a negative sense, single-stranded RNA virus belonging to the *paramyxoviridae* family. RSV leads to acute lower respiratory tract infection and causes several symptoms, such as wheezing, cough, fever, and severe bronchiolitis in infants, immunocompromised individuals, and the elderly. Moreover, 2–3% of infants who are infected with RSV require hospitalization owing to disease severity [1]. RSV is divided into two major subtypes, RSV A and B, depending on the sequence of attachment of the (G) glycoprotein [2, 3]. According to reports, both RSV subtypes co-circulate alternately at 1-2-year intervals during each RSV epidemic [4], and > 60% of infants are infected during their first RSV season, and moreover most children who are exposed to RSV in their early life experience secondary RSV infection [5]. Repeated natural RSV infections occur throughout life owing to an absence of long-term immunity against RSV subtypes [6]. Because of repeated infections and the high risk to infants, it is necessary to develop an RSV vaccine which can counteract both RSV subtype infections. There is as yet no authorized vaccine for human use.

Near the end of the 1960s, a formalin-inactivated RSV (FI-RSV) vaccine based on the RSV A subtype was developed and tested in clinical trials in infants and young children [7]. However, upon subsequent natural RSV infection, FI-RSV did not protect against RSV infection and respiratory diseases were exacerbated. According to some reports, these phenomena were probably due to increase vaccine-induced immune-pathological effects, including pulmonary eosinophilia, which were caused by exaggerated Th2 type CD4^+^ T-cell responses [8–12]. Also, immunization with recombinant vaccinia virus expressing the RSV G protein (vvG) showed similar results in a mouse model [13–15]. For these reasons, RSV vaccine development requires a particular concentration on safety to prevent vaccine-enhanced diseases.

The RSV attachment (G) glycoprotein is an envelope protein with the RSV fusion (F) protein, which mediates attachment to target cells. The G protein is a major protective antigen that can induce a strong neutralizing antibody, indicating that the G protein is a key target protein for RSV vaccine development. According to previous reports, the RSV G protein has a great deal of antigenic and genetic variability, and the amino acid sequence similarity is only 53% between RSV A and B [2, 3]. However, there is a central conserved region (a.a. 155–206) of the G protein that contains the following features: first, a highly conserved amino sequence (a.a.164-176) that exists in all RSV subtypes [16]. Second, five independent B cell protective epitopes, which can induce epitope-specific antibody responses to protect against RSV infection [17]. Third, as the central conserved region of the G protein involves a CD4^+^ T-cell epitope (a.a. 183–195), it can induce epitope-specific CD4^+^ T cell immune responses [18]. And, last, the RSV G protein contains a CX3C chemokine motif (a.a. 182–186) in the central conserved region [19]. The CX3C motif can interact with CX3CR1, which is expressed by immune cells such as monocytes, macrophages, T cells, and NK cells, and modulate innate and adaptive immune responses. Recent reports indicate that an RSV G polypeptide involving the CX3C motif elicited IgG antibodies which blocked the G protein-CX3CR1 interaction [20–22]. Thus, if CX3C motif-specific antibodies are induced, they may be able to reduce the immunopathology and immunomodulation following RSV G protein-CX3CR1 interaction. Therefore, these features of the RSV G protein can provide an advantage in RSV vaccine development.

The most common respiratory viral pathogens infect mucosal surfaces in the respiratory tract. Thus, mucosal immune responses are generally the first line of defense against such pathogens [23, 24]. In order to elicit protective mucosal immunity, immunization through mucosal routes, such as nasal or sublingual routes, may be one of the effective methods compared to non-mucosal routes [23]. Mucosal immunization induces mucosal antibodies as well as systemic antibodies, and elicits systemic CTL responses when antigens are administered with adjuvants such as cholera toxin (CT) [25]. Thus, administration through mucosal routes can effectively defend against invasion of respiratory pathogens into the host mucosa. Among the mucosal routes, since immunization via the intranasal route can elicit stronger systemic and mucosal antibody responses than is possible with other mucosal routes [23], the intranasal route is considered to be appropriate for administration of vaccines against respiratory pathogens. However, this route involves safety concerns owing to neurological side effects which are caused by retrograde transport of antigens or adjuvants via the olfactory epithelium [26]. Sublingual immunization also induces both systemic and mucosal antibody responses, while avoiding the safety issues associated with intranasal administration.

Our group has studied the effects of mucosal immunization with the RSV G core fragment. A single intranasal immunization with recombinant adenovirus-based vaccine encoding the central core region (a.a. 130–230) of the G protein from the RSV A subtype (rAd/3×G) induced humoral immune responses including serum IgG and mucosal IgA, and protected against RSV A subtype infection. Interestingly, rAd/3×G administration via intranasal route also partially protected against RSV B subtype infection [27]. Immunization with the Gcf subunit vaccine, which consists of the RSV G core fragment (a.a 131–230) and CT as an adjuvant, also induced humoral immune responses, especially when administered intranasally; intranasal administration induced a greater mucosal IgA response than did intramuscular injection [28]. Furthermore, sublingual immunization with wtBGcf, which consists of the G core fragment (a.a. 131–230) from RSV B1, elicited RSV B G-specific humoral antibody responses without inducing eosinophil recruitment upon RSV B subtype infection [29].

Based on these results, we generated a fusion protein, GcfAB, which consists of the central regions (a.a residues 131–230) of RSV G from both the RSV A and RSV B1 subtypes and investigated the humoral and adaptive immune responses generated by intranasal (IN) or sublingual (SL) immunization with the novel GcfAB vaccine with cholera toxin (CT) as an adjuvant. Then, we evaluated the protective efficacy against RSV A and RSV B infection according to different administration route. Our results showed that GcfAB-immune mice inoculated via the intranasal route expressed more RSV G-specific humoral immune responses than did those inoculated via the sublingual route. Also, GcfAB immunization via both mucosal routes protected from RSV A and B subtype infection. However, vaccine-enhanced immunopathology, such as eosinophil recruitment and body weight loss, was induced following sublingual immunization compared with intranasal immunization. Overall, these results indicate that mucosal immunization with GcfAB efficiently induces broad protective immunity against both RSV subtypes, but sublingual immunization elicits more vaccine-enhanced immunopathology than does intranasal immunization.

## Materials and methods

### Construction and purification of a recombinant fusion protein consisting of G core fragments from the RSV A and B subtypes

We designed a recombinant fusion protein, GcfAB, using the pET-21d-GcfA and pET-21d-GcfB expression plasmid, which have been previously described [28, 29]. The coding sequence of the RSV B G protein from amino acid residues 131–230 of the RSV B1 subtype was amplified with the forward primer (5’-AAA AGC TTA CAA CCG CCC AGA CC-3’) and the reverse primer (5’-CCC TCG AGG GGG TTT GTG GTT GTT-3’) by PCR using the pET-21d-GcfB expression plasmid as the template. The GcfB target DNA fragment was ligated into the Hind III and Xho I sites of the pET-21d-GcfA plasmid (pET-21d-GcfAB).

The pET-21d-GcfAB plasmid was transformed into *E*. *coli* BL21 (DE3) and cultured overnight in LB medium containing 50 μg/ml ampicillin at 37°C. The overnight culture was transferred into fresh LB medium containing 50 μg/ml ampicillin and grown until it reached an OD600 of 0.5–0.7 at 37°C with shaking. Overexpression was induced by adding 1mM isopropyl β-D-1-thiogalactopyranoside (IPTG) at 37°C for 4 hr. Then, the bacterial pellets were harvested by centrifugation at 6,000 rpm for 20 min. The cells were resuspended in binding buffer (20 mM KPO_4_, 0.5 M NaCl, 10 mM imidazole, pH 7.4) and disrupted by sonication on ice. The disrupted bacterial cells were centrifuged at 15,000 rpm for 30 min, and the supernatant was collected and loaded onto a His Trap affinity column (GE Healthcare), which was washed with binding buffer. Loaded proteins were eluted using an elution buffer (20 mM KPO_4_, 500 mM NaCl, 500 mM imidazole, pH 7.4). Eluted target protein fractions were collected, concentrated, and buffer-exchanged with PBS using a centrifugal filter device (Centricon 15 ml, 50 ml, 10 kDa MWCO, Millipore, USA). The concentrated target protein samples were loaded onto a HiPrep 16/60 Sephacryl S-200 HR (GE Healthcare) column equilibrated with PBS. After collection of the purified monomer peak fractions, contaminated endotoxins were removed using 1% Triton X-114. The endotoxin levels in the purified protein samples were measured by a Pierce LAL chromogenic endotoxin quantitation kit (Thermo, USA). The endotoxin level of the protein was less than 5 EU/mg. The purified proteins were subjected to 12% SDS-PAGE and visualized by staining with Coomassie Brilliant Blue (CBB). The concentration of purified protein was determined by a Pierce BCA protein assay kit (Thermo, USA) and stored at –80°C until further use.

### Virus preparation

The RSV A2 and B (human RSV-B isolate, KR/B/10-12 [27]) strains were propagated in HEp-2 cells (ATCC, Manassas, VA) using MEM (Welgene, South Korea) supplemented with 3% heat-inactivated FBS, 2 mM glutamine, and 20 mM HEPES. When extended syncytia were observed in infected cells, on about day 4 post-infection, viruses were harvested and concentrated by ultra-centrifugation. The pellets were resuspended in serum-free MEM using a 25-gauge needle and sonication. Virus titer was determined using a standard RSV plaque assay and the viruses were stored at –80°C until use.

### Mice and ethics statement

6-8-week-old female BALB/cAnNCrljOri mice were purchased from Orient Bio Inc. (Seoul, Republic of Korea) and housed under specific pathogen-free conditions. All animals were monitored daily and handled gently in order to reduce stress and alarm. And all animal experiments were performed strictly in accordance with the suggestions in the Institute of Laboratory Animal Resources Guide for the Care and Use of Laboratory Animals. Also, this study was approved by the guidelines of Ewha Womans University Institutional Animal Care and Use Committee (IACUC, Approval Number. 15–073).

### Immunization and viral challenge in mice

Mice (n = 4/group) were immunized with PBS (in 50 μl) as a negative control, 20 μg of purified GcfAB, GcfA or GcfB proteins with 2 μg of cholera toxin (CT; List Biological Laboratories, Campbell, CA) via intranasal (IN, in a final volume of 50 μl) or sublingual (SL, in a final volume of 15 μl) routes twice, on days 0 and 18. For IN immunizations, mice were anesthetized by isoflurane (Ifran^®^; Hana Pharm, Korea) inhalation, and antigen or virus was delivered to the left nostril. For SL immunizations, mice were anesthetized by i.p. injection of 100 mg/kg body weight ketamine (Yuhan Co., Seoul, Korea) and 10 mg/kg body weight Rompun (Bayer Korea, Seoul, Korea) mixture in PBS, and antigen was gently delivered underneath the tongues of the mice. For a more reliable sublingual delivery, mice were maintained with their heads placed in ante flexion for at least 30 minutes. Recombinant vaccinia virus expressing the attachment glycoprotein of RSV (vvG) as a positive control, was inoculated by skin scratch with a 25-gauge needle at the base of the tail (5×10^6^ PFU in 10 μl) once. For tail scratch, mice also were anesthetized by i.p. injection of ketamine/Rompun mixture in PBS as described above, and after scratching the tail, 10 μl of prepared vvG was absorbed on the scratched skin. At 3–4 weeks after the last immunization, the mice were challenged with RSV A2 (1×10^6^ PFU in 50 μl) or KR/B/10-12 (RSV B subtype; 2–4×10^6^ PFU in 50 μl) through IN route. To investigate a body weight loss, the challenged mice were monitored daily for 5 days, and when mice had lost ~ 25% of their maximum body weight or were observed such as decreased activity, lack of grooming, or hypothermia, we planned to sacrifice the mouse by CO_2_ euthanasia. In the RSV A2 challenge study, GcfAB-immune groups showed clinical signs such as decreased activity, lack of grooming, and/or body weight loss (GcfAB SL group had lost 20% of their maximum body weight), but not serious enough to end animal experiments.

### Bronchoalveolar Lavage (BAL)

Five or 7 days after viral challenge, mice were sacrificed by CO_2_ euthanasia and bronchoalveloar lavage (BAL) fluids were collected by washing the airways with 1 ml of PBS. BAL cells and supernatants were separated via centrifugation, and the collected cells and supernatants were used to determine leukocyte recruitment and secretory IgA levels.

### ELISA

To measure RSV G-specific antibody responses, such as IgG and IgA, we performed direct enzyme-linked immunosorbent assay (ELISA). Briefly, ELISA plates (NUNC maxisorp, Thermo Scientific) were coated with 50 ng/well of purified G core fragment of RSV A2 (GcfA) or 100 ng/well of purified G core fragment of RSV B1 subtype (GcfB) in 100 μl of PBS overnight at 4°C. Each antigen-coated well was blocked with PBS containing 1% non-fat milk for 2 hr at room temperature (RT) and washed with PBS containing 0.05% Tween-20. Then, each serum or BAL fluid sample was serially diluted in blocking buffer and incubated for 2 hr at RT. The plates were washed, HRP-conjugated goat anti-mouse IgG (Abcam) or HRP-conjugated goat anti-mouse IgA (Zymed Laboratories, San Francisco, CA) were added to the appropriate wells, and the samples were incubated for 1 hr at RT in the dark. After incubation, the plates were washed six times and tetramethylbenzidine peroxidase substrate (TMB, KPL, Gaithersburg, MD) was added to develop the color. The reaction was stopped by 1 M H_3_PO_4_, and the optical density (OD) was measured at a wavelength of 450 nm using a Thermo ELISA plate reader.

In order to detect epitopes of RSV G-specific IgG antibody responses, streptavidin-coated ELSIA plates were washed and 200 ng/well of biotinylated G peptides, such as residues 144–159, 164–176, 174–187, and 190–204 of RSV A2, were added for 3 hr at RT. After incubation, we performed the assay as stated above.

### Flow cytometric analysis

Immunized mice were sacrificed by CO_2_ euthanasia and BAL cells and lung lymphocytes were collected at 5 or 7 days after viral challenge. To measure granulocytes, BAL cells were incubated for 10 min with rat anti-mouse CD16/CD32 (BD biosciences, California, USA) blocking antibody at RT and then stained with anti-Gr-1(RB6-8C5), anti-Siglec-F (E50-2440), anti-CD11c (N418), and anti-CD45 (30-F11) antibodies for 30 min at 4°C. After staining, the cells were fixed in FACS lysing solution (BD biosciences) and acquired using a FACS Calibur flow cytometer (BD biosciences). To investigate the cytokine-producing cells, the lung tissues were homogenized and passed through a 70 μm cell strainer (SPL). After centrifugation, the cells were resuspended in IMDM supplemented with 10% heat-inactivated FBS and stimulated with 50 μg/ml PMA (1:1000) and 500 μg/ml ionomycin (1:1000) or 10 μM RSV G peptide (a.a. 183–195 of RSV A2 and B1 strains) for 5 hr in the presence of Brefeldin A (eBioscience) at 37°C. After stimulation, the surface markers of these cells were stained with anti-CD3 (17A2) and anti-CD4 (RM4-5) for 30 min at 4°C and then fixed and permeabilized with FACS buffer (0.5% FBS, 0.09% NaN3 in PBS) containing 0.5% saponin (Sigma-Aldrich) for 15 min at RT. The IFN-γ and IL-17A cytokines were stained with anti-IFN-γ (XMG 1.2) and anti-IL-17A (TC11-18H10.1) at RT and then acquired using a FACS Calibur flow cytometer (BD biosciences) and a BD LSR Fortessa (BD biosciences). All flow cytometry data was analyzed using Flowjo software (TreeStar Inc., Ashalend, OR, USA).

### Viral titer in the lungs

Five days after RSV A (A2) or B (KR/B/10-12) subtype challenge, the mice were sacrificed by CO_2_ euthanasia, and lung tissues were harvested and processed through a 70 μm cell strainer (SPL) into serum-free MEM. After centrifugation, the supernatant was collected and RSV titer was measured by a standard plaque assay in HEp-2 cells using the supernatants. The viral titer is expressed as PFU/g of lung tissue; the limit of detection is 100 PFU/g.

### Histology

To investigate lung histology, mice were sacrificed at 5 days post-RSV A2 challenge and lungs were harvested after perfusion. Harvested lungs were fixed in 4% formalin for 48 hours, embedded in paraffin, and sectioned. Lung sections were stained with hematoxylin and eosin (H&E) or periodic acid-Schiff (PAS) and evaluated by light microscopy (10× magnification).

### Statistical analysis

All data are expressed in mean ± SD (n = 4/group). Statistical differences were analyzed by an unpaired, two-tailed Student’s t-test. A P value ≤ 0.05 was taken to indicate statistical significance.

## Results

### Construction and purification of the GcfAB subunit vaccine

**Fig 1:**
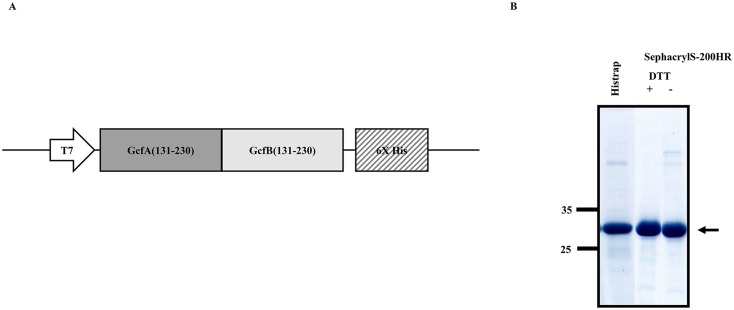
Expression and purification of recombinant GcfAB protein. Schematic diagram of the GcfAB recombinant protein. (A) A pET-21d plasmid containing both a.a 131 to 230 of RSV A2 G protein (GcfA) and a.a. 131 to 230 of RSV B1 G protein (GcfB) was constructed. (B) The GcfAB protein was purified by His-tag affinity chromatography (lane 1) and size-exclusion chromatography (Sephacryl S-200 HR) with (lane 2) / without (lane 3) dithiothreitol (DTT). Purification of GcfAB was confirmed by SDS-PAGE at each purification step, as indicated by the arrow.

To develop the dual subunit vaccine covering both subtypes (A and B) of RSV, we employed a strategy that fuses a highly-conserved central region (a.a. 131–230) of the RSV A2 G sequence with that of the RSV B1 G sequence, resulting in a GcfAB fusion in the pET-21d vector (Fig 1A). It was previously shown that a.a. residues 183–195 of GcfA functions as a CD4 T-cell epitope that is necessary for induction of a strong antibody response, while the same region of GcfB lacks T-cell epitope functionality and induces a relatively weak antibody response [29]. Thus, we reasoned that fusion of the GcfA sequence to the GcfB sequence might simultaneously provoke T-cell activity against both GcfA and GcfB, and thus, that immunization with the GcfAB fusion antigen might induce strong humoral responses against both subtypes. To this end, the GcfAB protein was purified from *E*.*coli* by His-tag affinity chromatography and size-exclusion chromatography, and its purity was confirmed using SDS-PAGE. The approximate molecular weight of the monomeric form of GcfAB is ~33 kDa (black arrow) on SDS-PAGE under reducing conditions (Fig 1B).

### Humoral antibody responses in GcfAB-immune mice

**Fig 2:**
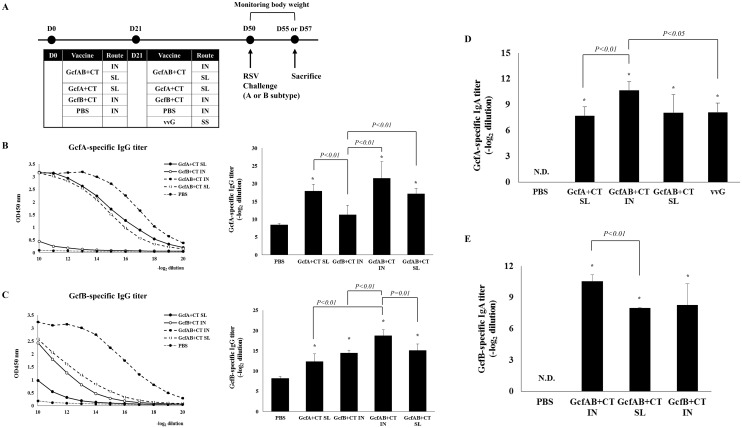
Characterization of humoral immune responses in GcfAB-immune mice. (A) The scheme used for animal experiments is shown in Fig 2. (B and C) Twenty-one days after the last immunization, RSV G-specific IgG titers in sera were measured by ELISA. GcfA (50 ng/well) or GcfB (100 ng/well) proteins were used as coating antigens, and goat anti-mouse IgG-HRP was used as a detection antibody. The cutoff optical density at 450 nm (OD 450 nm) was < 0.15 for a PBS negative result. (D and E) Five days after RSV A2 or B (KR/B/10-12) challenge, BAL fluid was harvested and the GcfA-specific or GcfB-specific mucosal IgA antibody responses were measured by ELISA. The results represent log_2_ endpoint values averaged from four mice. N.D., not detected. All data are expressed in mean ± SD (n = 4/group). Significant differences from the PBS control group are marked with asterisks (*). *p < 0*.*01*.

In order to investigate whether mucosal GcfAB immunization can elicit an antibody responses against both RSV A and B subtypes, BALB/c mice were immunized twice via intranasal (IN) or sublingual (SL) routes with 20 μg of GcfAB plus 2 μg of CT as a mucosal adjuvant (Fig 2A). Twenty-one days after immunization, RSV G-specific serum IgG antibody responses were measured by ELISA. Both the GcfAB IN and SL groups exhibited significant subtype A-specific serum IgG responses compared to the negative control PBS group (Fig 2B), but statistically significant differences between GcfAB IN and SL groups were not found (p = 0.1). The GcfAB IN and SL groups also exhibited subtype B-specific serum IgG responses (Fig 2C); GcfAB IN group induced significantly higher responses than both GcfB IN and GcfAB SL groups (p < 0.01), but GcfAB SL group did not differ significantly from the GcfB IN group (p = 0.4). The GcfA SL group and the GcfB IN group exhibited subtype-specific IgG responses (Fig 2B and 2C).

We next analyzed the secretory IgA antibody response following mucosal GcfAB vaccination. Mucosal IgA is important in the defense against respiratory virus infections, and is also associated with protective immunity against RSV infection [30]. To this end, GcfAB-immunized mice were challenged with RSV A (A2) or B (KR/B/10-12). GcfA SL and vvG were used as a positive controls for RSV A2 challenge and GcfB IN was used as a positive control for RSV B challenge. At 5 days post-challenge, the RSV A (Fig 2D) or RSV B (Fig 2E) subtype-specific mucosal IgA responses were measured by ELISA using bronchoalveolar lavage (BAL) fluid collected from each group. GcfA SL group induced RSV A-specific IgA response, and vvG group also induced RSV A-specific IgA response (Fig 2D). The levels of GcfA- or GcfB-specific mucosal IgA were significantly higher in the GcfAB IN and SL groups than PBS group (Fig 2D and 2E). Especially, GcfAB IN group elicited significantly higher GcfB-specific mucosal IgA response than GcfAB SL group against RSV B challenge (Fig 2E). The GcfB IN group also induced GcfB-specific mucosal IgA response, but it was not statistically different among the GcfAB-immune groups (Fig 2E). There was no detectable level of mucosal IgA antibody in the PBS group (negative control) against both RSV A and RSV B. Taken together, these results show that GcfAB immunization via either IN or SL routes effectively induces both RSV subtype-specific serum IgG and mucosal IgA antibody responses.

### Epitope-specific antibody responses were induced by GcfAB immunization

**Fig 3:**
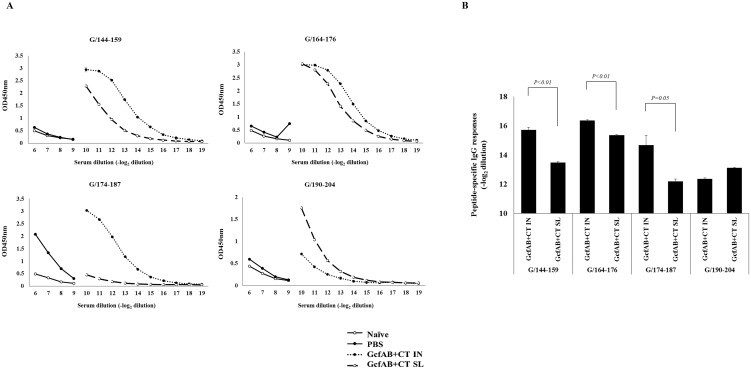
Epitope-specific antibody responses against RSV G peptides in GcfAB-immune mice. BALB/c mice (n = 4/group) were immunized via intranasal or sublingual routes with 20 μg of GcfAB and 2 μg of CT. (A and B) After a booster immunization, peptide-specific IgG titers were determined in mouse sera. Biotinylated G peptides (a.a. 144–159, 164–176, 174–187 and 190–204 of the RSV A2 subtype) were used as a coating antigen (200 ng/well), and goat anti-mouse IgG-HRP was used as a detection antibody.

Previous studies have shown that the RSV G protein contains several linear B cell epitopes [19, 31]. As these epitopes are present in the central conserved region of the RSV G protein [17], we expect that GcfAB can induce a variety of epitope-specific antibody responses. We investigated the epitope-specific antibody responses following GcfAB mucosal immunization. We performed ELISA using biotinylated peptides spanning residues 144–159, 164–176, 174–187 [32], and 190–204 of RSV A2 (Fig 3). The GcfAB IN group induced higher G/144-159 and G/164-176 peptide-specific serum IgG responses than did the GcfAB SL group (Fig 3A and 3B). Notably, G/174-187-specific serum IgG response was significantly higher in the GcfAB IN group than in the GcfAB SL group, in which the response was barely detectable above background levels. However, G/190-204-specific serum IgG response was reversed; GcfAB SL group showed higher G/190-204-specific antibody response than that of GcfAB IN group (Fig 3A) but there was no significant difference (Fig 3B). These results indicate that the antibody responses to G/164-176 peptide are dominantly induced both in GcfAB IN and SL groups, and IN immunization generally elicits higher levels of all epitope-specific antibody responses except for G/190-204 peptide than in SL immunization. By contrast, G/190-204-specific antibody response was generally weaker than other peptides-specific responses. Furthermore, we can expect that the types of epitope-specific antibody responses may be different depending on the administration routes, even with the same antigen.

### RSV A-specific CD4 T-cell responses are induced in GcfAB-immune mice after RSV A2 challenge

**Fig 4:**
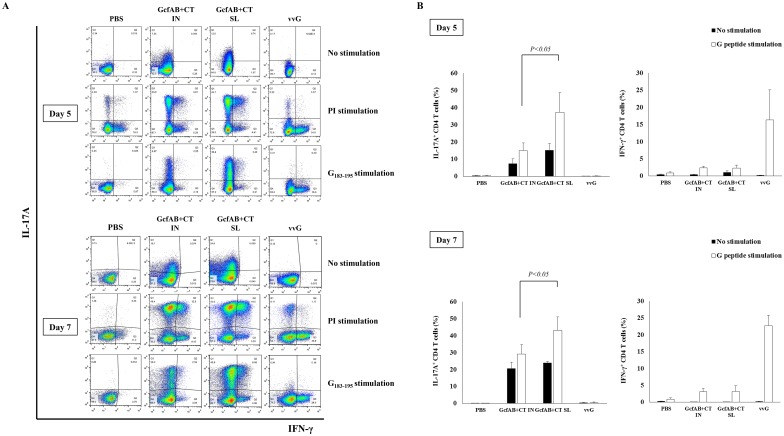
RSV A-specific Th1 and Th17-cell responses in GcfAB-immune mice after RSV A challenge. Five and 7 days after RSV A2 challenge, lung mononuclear cells were isolated from GcfAB-immune mice. Cells were stimulated with PMA (50 μg/ml) and ionomycin (500 μg/ml) as a positive control, or with/without RSV G (a.a. 183–195 of RSV A2 subtype) peptide for 5 hours. Lung cells were stained for anti-CD3, CD4, IFN-γ, and IL-17A antibodies, and were analyzed by flow cytometry. (A) Cells gated for CD3^+^ CD4^+^ are shown in dot plots, (B) and the percentage is represented as the frequency of RSV G-specific IFN-γ^+^ and IL-17A^+^ CD4 T cells. Data are represented in mean ± SD (n = 4/group). Significant differences from the PBS group are marked with asterisks (*, **). *p < 0*.*05*.

To analyze whether mucosal GcfAB vaccination primes RSV G-specific CD4^+^ T-cell responses, GcfAB-immune mice were challenged with 1×10^6^ PFU of RSV A2 and lung cells were harvested 5 and 7 days post-challenge. Lung lymphocytes were stimulated with the G/183-195 peptide of the RSV A subtype (WAICKRIPNKKPGKK) for 5 hours *ex vivo*, and the levels of both IFN-γ and IL-17A were measured by flow cytometry (Fig 4A). After G/183-195 peptide stimulation, GcfAB-immune groups induced both IFN-γ^+^ (~ 3% of total lung CD4 T cells on average) and IL-17A^+^ CD4 T cells (25% ~ 40% of total lung CD4 T cells on average) compared to the PBS group (Fig 4B). Interestingly, GcfAB SL group showed a significantly higher IL-17A^+^ CD4 T-cell response than did the GcfAB IN group (Fig 4B). The vvG group as a positive control, showed strongest IFN-γ^+^ CD4 T-cell response (about 20% of total CD4 T cells on average), but did not exhibit noticeable IL-17A^+^ CD4 T-cell response (Fig 4B). In contrast, GcfAB-immune groups induced relatively weak IFN-γ^+^ CD4 T-cell responses at 5 and 7 days post-challenge, compared with vvG group. Meanwhile, both the GcfAB IN and SL groups showed a similar responses in IFN-γ^+^ and IL-17A^+^ double-positive CD4 T cells (data not shown). This result indicates that mucosal GcfAB immunization induce Th17-type dominant CD4^+^ T-cell responses, and noticeably, SL immunization elicited stronger Th17-cell responses than did IN immunization.

### CD4 T-cell responses for RSV B in GcfAB-immune mice after RSV B challenge

**Fig 5:**
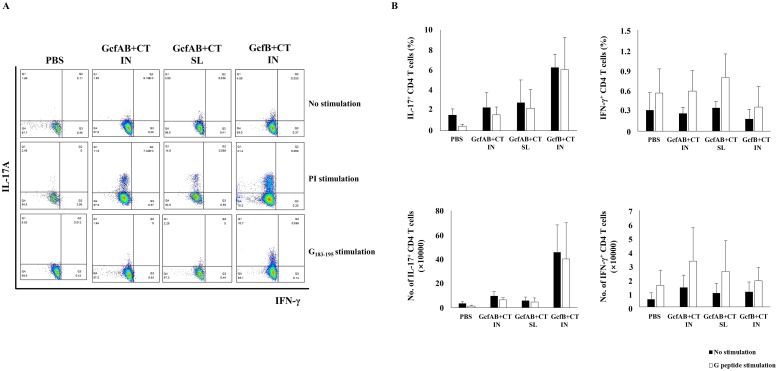
RSV B-specific Th1 and Th17-cell responses in GcfAB-immune mice after RSV B challenge. After RSV B (KR/B/10-12) challenge, all mice (n = 4/group) were sacrificed and lung mononuclear cells were isolated 5 days post-infection. Lung lymphocytes were stimulated and were analyzed as the same manner as in Fig 4. (A) CD3^+^ and CD4^+^ cells are shown in the dot plots, (B) and the percentage of such cells is represented as the frequency of RSV G-specific IFN-γ^+^ and IL-17A^+^ CD4 T cells. Data are represented in mean ± SD (n = 4/group). Significant differences are marked with a hash tag (#, *p < 0*.*05*).

Next, in order to investigate whether GcfAB elicits CD4 T-cell responses upon RSV B subtype infection, GcfAB-immune mice were challenged with an RSV B (KR/B/10-12) clinical isolate with the same 131–230 amino acid sequence as GcfB. Five days post-challenge, GcfAB-immune mice were sacrificed and lung lymphocytes were stimulated with the G/183-195 peptide of the RSV B subtype (KSICKTIPSNKPKKK) which corresponds to the CD4^+^ T-cell epitope of GcfA. After 5 hours, the levels of IFN-γ- and IL-17A-expressing CD4 T cells were measured by flow cytometry (Fig 5A) in the same manner as in Fig 4. Both IFN-γ- and IL-17A-expressing cells were significantly fewer following RSV B G peptide stimulation (Fig 5B) as compared to those seen following RSV A2 G peptide stimulation after RSV A challenge (Fig 4B). These results indicate that a.a. residues 183–195 of the RSV B G protein do not play roles as CD4^+^ T-cell epitopes, as previously reported [29]. Consequently, CD4 T-cell responses for the RSV B subtype were not found in our study.

### Protective efficacy of GcfAB vaccine against both RSV A and B subtype infections

**Fig 6:**
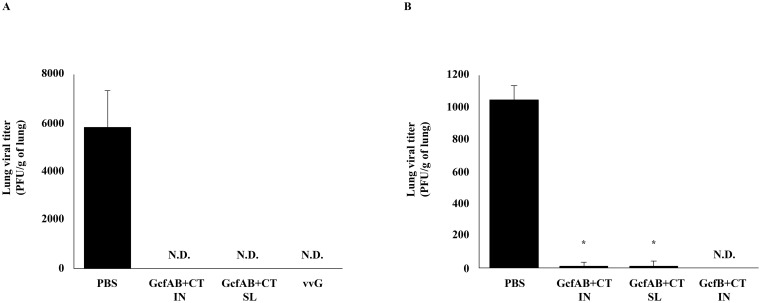
GcfAB-immune mice inoculated via intranasal or sublingual routes are protected from both RSV A and B subtype infections. All mice (n = 4/group) were challenged with (A) 1×10^6^ PFU/mouse of RSV A2 or (B) 2–4×10^6^ PFU/mouse of RSV B (KR/B/10-12). Five days post-challenge, lung viral titer was determined by a plaque assay. The limit of detection is 100 PFU/g of lung tissue. N.D., not detected. Data are represented in mean ± SD (n = 4/group). Significant differences from the PBS group are marked with asterisks (*), *p < 0*.*01*.

Since GcfAB immunization via different mucosal routes (IN and SL) induced significant humoral and cellular immune responses, we next investigated whether mucosal GcfAB immunization provides protection against both subtype infections. To this end, GcfAB-immune mice were challenged with RSV A (A2 strain) or RSV B (KR/B/10-12 strain), and the efficacy was measured by performing a standard plaque assay with lung samples 5 days post-challenge (Fig 6). As shown in Fig 6, GcfAB immunization protected mice against both RSV A (Fig 6A) and RSV B subtype (Fig 6B) challenge. vvG (as a positive control for RSV A subtype challenge) and GcfB+CT (as a positive control for RSV B subtype challenge) defended against each subtype. This result indicates that GcfAB immunization via both the IN and SL routes can provide protection against both the RSV A and B subtypes.

### Vaccine-enhanced pulmonary cell infiltration in GcfAB-immune mice after both RSV A and B subtype challenge

**Fig 7:**
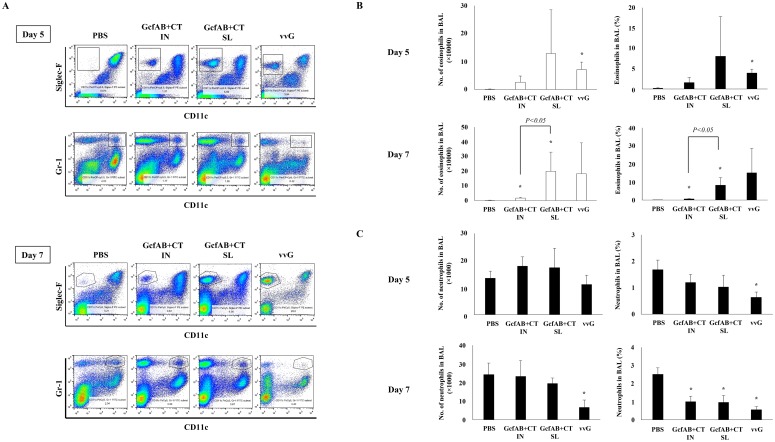
Pulmonary cell infiltration in GcfAB-immune mice following RSV A subtype challenge. After two rounds of GcfAB vaccine administration, the GcfAB-immune groups were challenged with RSV A2 and then sacrificed at 5 and 7 days post-challenge. (A) BAL cells were harvested and then stained with anti-CD45, Siglec-F, Gr-1, and CD11c antibodies and analyzed by flow cytometry. (B and C) Eosinophils and neutrophils among CD45^+^-gated cells were quantitated. All data represented in mean ± SD (n = 4/group). Significant differences from the PBS group are marked with asterisks (*). *p < 0*.*05*.

**Fig 8:**
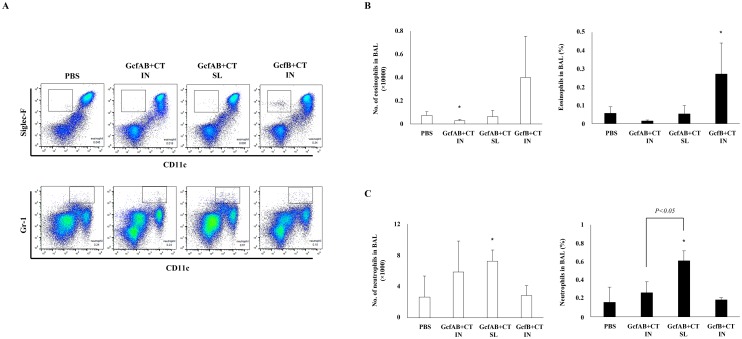
Low level of pulmonary cell infiltration in GcfAB-immune mice after RSV B subtype challenge. After two rounds of GcfAB vaccine administration, GcfAB-immune mice were challenged with RSV B (KR/B/10-12) and sacrificed for analysis 5 days post-challenge. (A) BAL cells were harvested and stained with anti-CD45, Siglec-F, Gr-1, and CD11c antibodies and then analyzed by flow cytometry. (B and C) Eosinophils and neutrophils among CD45^+^- gated cells were quantitated. Data are expressed in mean ± SD (n = 4/group). Significant differences from the PBS group are marked with asterisks (*). *p < 0*.*05*.

According to previous reports, RSV G-expressing vaccine candidates, such as vvG or subunit vaccines, consisting in part of the RSV G protein, showed excessive pulmonary eosinophilia following live RSV infection [14, 15, 18]. To investigate whether GcfAB immunization leads to pulmonary cell infiltration, GcfAB-immune mice were challenged with either RSV A2 (Fig 7) or RSV B (KR/B/10-12) (Fig 8). Five and 7 days post-challenge, we measured the percentages of both eosinophils and neutrophils in bronchoalveolar lavage (BAL) cells by flow cytometry (Figs 7A and 8A). After RSV A subtype challenge, the GcfAB SL group exhibited greater recruitment of eosinophils than did the GcfAB IN group, and the number of eosinophils was increased at 7 days compared to 5 days (Fig 7B). The vvG immune group (positive control) showed a significantly increased number and percentage of eosinophils, while the PBS group did not exhibit any detectable eosinophil recruitment (Fig 7B). The percent of neutrophils was significantly reduced in the GcfAB-immune groups compared with the PBS group at 7 days (Fig 7C), but both the percentage and number of neutrophils were not significantly different between the PBS and GcfAB-immune groups (p > 0.05) at 5 days (Fig 7C).

Meanwhile, following RSV B subtype challenge, overall, both the numbers and percentages of eosinophils and neutrophils were lower than those of the GcfAB-immune groups challenged with the RSV A subtype. The GcfAB-immune groups showed a low level of eosinophils (< 0.1% of CD45 cells), but the GcfB IN group showed high level of eosinophil recruitment compared with the GcfAB-immune groups (Fig 8B). Neutrophils were induced in approximately a 3-fold greater extent in the GcfAB SL group than in the GcfAB IN group (Fig 8C). Taken together, these data indicate that SL immunization with GcfAB elicits significantly more vaccine-induced eosinophil infiltration than does IN immunization following RSV A challenge. On the contrary, GcfAB immunization induces low-level pulmonary cell infiltration upon RSV B subtype challenge, which probably leads to a very weak inflammatory response in the airways.

### Vaccine-enhanced disease in GcfAB-immune mice after RSV A and B subtype challenge

**Fig 9:**
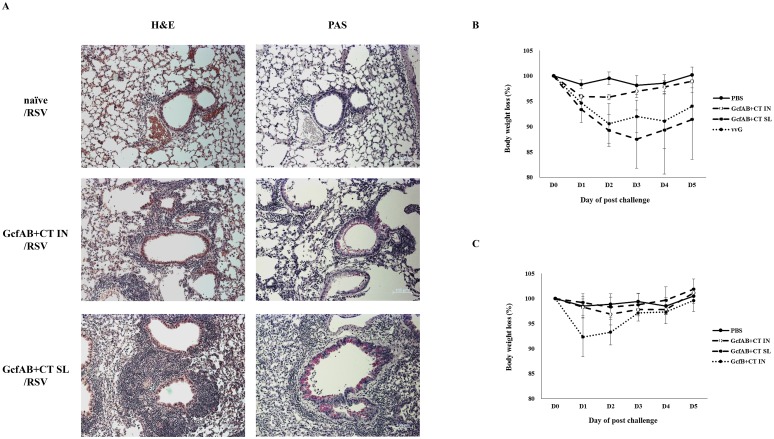
Vaccine-enhanced disease in GcfAB-immune mice after RSV A or B subtype challenge. To investigate cellular infiltration and mucus secretion in the lungs, we performed the (A) H&E staining (left column) and PAS staining (right column) of lung tissues which were harvested at 5 days post-RSV A2 challenge, and evaluated by light microscopy (10× magnification). Scale bar, 100 μm. Body weight was monitored for 5 days post-challenge with (B) 1×10^6^ PFU of RSV A2 or (C) 2–4×10^6^ PFU of RSV B (KR/B/10-12). Data are expressed in mean ± SD (n = 4/group).

We determined that GcfAB immunization provoked a different amount of vaccine-induced cellular infiltration in BAL (Fig 7) and lung tissue (Figs 4 and 5) depending on mucosal immunization route and subtype infection. So, we investigated whether the use of differential immunization routes results in any differences in vaccine-induced disease patterns following RSV subtype challenge. We have identified RSV A but not RSV B challenge causes pulmonary cell infiltration in GcfAB-immune mice (Figs 7 and 8). In order to determine the histopathology in the lung, we performed H&E and PAS staining on the lung tissues which were harvested at day 5 post-RSV A2 challenge (Fig 9A). Consistent with flow cytometric data, both GcfAB IN and SL groups induced inflammatory cell infiltrations compared to PBS group in the RSV A2 challenge. Noticeably, GcfAB SL group elicited higher degree of inflammatory cell infiltration than GcfAB IN group (Fig 9A, left column, 10× magnification). Higher degrees of airway mucus secretion and goblet cell hyperplasia were also induced in GcfAB SL group than in GcfAB IN group (Fig 9A, right column, 10× magnification). GcfAB-immune mice were challenged with RSV A2 or RSV B (KR/B/10-12) and body weight loss was monitored for 5 days after RSV challenge (Fig 9B and 9C). Following RSV A2 challenge (Fig 9B), the GcfAB SL group and the vvG group experienced significant weight loss compared with the PBS group. The GcfAB SL group showed rapid and severe weight loss until 3 days post-challenge, while body weight of the GcfAB IN group was slightly decreased at 1 day post-challenge and recovered from 2 days post-challenge onwards. Following RSV B subtype challenge (Fig 9C), however, both GcfAB-immune groups and the PBS group did not exhibit any significant decrease in body weight. The GcfB IN group showed a body weight decrease up to ~ 8% on day 1 (Fig 9C). Together, these data demonstrates that SL immunization with GcfAB causes massive inflammatory cell infiltration and severe body weight loss when compared with IN immunization after RSV A challenge, but not after RSV B challenge.

## Discussion

Respiratory syncytial virus is highly pathogenic and a serious cause of mortality in infants. Since the failure of the FI-RSV vaccine, many investigations into a safe RSV vaccine have been carried out, but RSV vaccines for human use are not yet available. In this study, we focused on the G attachment protein as the major target antigen for a vaccine, since G protein-specific immune responses are correlated with protection against RSV infection. We designed a universal vaccine composed of RSV G core fragments (a.a. 131–230) from both RSV A and B subtypes in order to elicit immunity against both subtypes. Also, we used two routes of mucosal administration, intranasal (IN) and sublingual (SL), in order to identify the mucosal immune responses that are most effective against respiratory virus infection.

We previously demonstrated that GcfA or GcfB-immune mice vaccinated via mucosal routes induced subtype-specific humoral immune responses involving serum IgG and mucosal IgA antibodies [28, 29]. Because GcfAB is composed of two RSV subtype G central domains, we expected to induce humoral immunity against both RSV subtypes in GcfAB-immune mice. As we predicted, GcfAB-immune mice successfully induced RSV A- and B-G-specific serum IgG and mucosal IgA antibody responses; in particular, the GcfAB IN group showed higher levels of antibody titers (both IgG and IgA) than did the GcfAB SL group. However, when we checked the *in vitro* neutralizing activity of serum antibody by plaque reduction neutralization assay (PRNA), GcfAB-immune groups did not show any noticeable neutralizing activity compared with PBS group in our study (data not shown). It is possible that RSV enters via carbohydrate moieties *in vitro* even when the CX3C-CX3CR interaction is blocked, in contrast to infection *in vivo*.

According to several serological studies, humoral responses against variable regions of the RSV G protein, such as universally conserved residues (a.a. 164–176) and/or the CX3C chemokine motif (a.a. 182–186), are associated with RSV immunity [16, 33, 34]. Since the RSV G central domain contains multiple B cell epitopes (a.a. 152–163, 165–172, 171–187, and 196–204) and each epitope is related with a protective immune response [17], we investigated antibody reactivity to each B cell epitope following mucosal administration of GcfAB. The results showed that the GcfAB IN group exhibited a relatively high level of serum antibody response against the G/144-159, G/164-176, and G/174-187 epitopes, but not the G/190-204 epitope, compared with the GcfAB SL group. In particular, G/174-187-specific antibody responses showed high reactivity in the GcfAB IN group compared with the GcfAB SL group. So, we posit that the mucosal route (IN and SL) of vaccine immunization may influence the immunodominance hierarchy for each B cell epitope-specific antibody. However, regardless of the different hierarchies of antibody responses, both the GcfAB IN and SL groups exhibited potential protection following RSV A and B challenge. Altogether, the results show that mucosal administration of GcfAB can induce mucosal antibody responses as well as systemic antibody responses against both RSV A and B, and are able to elicit antibody-mediated cross-protective immunity following infection with any RSV subtype. Although the two GcfAB-immune groups showed different antibody patterns for RSV G variable regions depending on mucosal route, the difference would not affect the protective efficacy following RSV infection.

The I^-^E^d^-restricted immune-dominant CD4 T-cell epitope (a.a. 184–198) of the RSV G central conserved region can elicit an epitope-specific CD4^+^ T-cell response [35–37]. It has been reported that CD4 T-cell responses play an important role in protection against RSV infection [38, 39]. However, RSV G-specific CD4 T-cell responses are also associated with immune-mediated pathology in RSV infection; for example, vvG- or FI-RSV vaccinated mice showed vaccine-induced immunopathology-mediated CD4^+^ T cell helper (Th1 and/or Th2) responses [14, 35, 40–42]. In this regard, we investigated representative Th1 and Th2 cytokine levels in BALF obtained from GcfAB-immune mice after RSV A challenge. Our groups previously showed that a Th2 cytokine response was not induced in GcfA/CT- or GcfB/CT-immune mice inoculated via mucosal routes, although these mice showed eosinophil infiltration in BAL [28, 29]. Th1 and Th2 cytokine responses were not biased towards one side; there is no significant difference between the GcfAB IN and GcfAB SL groups (data not shown). Thus, we concluded that GcfAB immunogen does not trigger Th2-biased responses.

In this study, we found that the GcfAB SL group exhibited more RSV-associated immunopathology, such as pulmonary eosinophilia, inflammatory cell infiltration, mucus secretion, and body weight loss, than did the GcfAB IN group following RSV A infection. When lung lymphocytes from the RSV-infected GcfAB IN and SL groups were restimulated with G/183-195 of the RSV A peptide, Th1 CD4^+^ T-cell responses were similarly induced in both the GcfAB IN and GcfAB SL groups, but these levels were lower than those in the vvG group. Interestingly, Th17 cell responses were strongly elicited in both GcfAB immunization groups but not vvG group; the GcfAB SL group showed a higher level of IL-17A^+^ CD4 T-cell response than did the GcfAB IN group after RSV A challenge. These results indicate that pulmonary eosinophilia is induced in both vvG and GcfAB immune groups, but the phenomenon seems to occur with different mechanisms; perhaps it may depend on cytokines which induce pulmonary eosinophilia. Previous study has reported STAT4 signaling pathway contributes to clinical diseases in vvG-immune mice following RSV challenge [43]. In our case, GcfAB-immune mice elicited excessive Th17 bias upon RSV challenge and Th2-type responses were not triggered; thus Th17 but not Th2-type responses might be associated with enhanced diseases appeared in this study. Recently, IL-17A-producing Th17 cells have been reported to be an important factor associated with immunopathology following RSV infection [44]. Several studies demonstrated that RSV-associated inflammatory responses, such as mucus production and airway hyperreactivity (AHR), were triggered by IL-17A [45, 46]. The Th17-skewed responses in our experiments are likely due to the cholera toxin (CT) adjuvant, given that we recently showed that CT induced Th17-dominated immune responses through an IL-6-dependant pathway following IN immunization [47]. Also, because GcfA without CT did not induce eosinophil accumulation in BAL [28], production of eosinophilia in GcfAB plus CT-immune groups may be related to Th17-cell responses. We are investigating the relationship between IL-17A and vaccine-induced immunopathology in G protein-based vaccine candidates with/without CT following RSV infection. One thing to note about these results is that we adopted the sublingual vaccination because it was considered safer than the intranasal route. However, in our case, it was found that the sublingual vaccination induced more severe immunopathology, suggesting that more careful investigation is needed when choosing sublingual vaccination.

The reason for the difference in vaccine-induced immunopathology by route of mucosal administration may be related to both antibody reactivity against the B cell epitope of the RSV G protein and the CX3C chemokine motif (a.a. 182–186), which is involved in the RSV G central conserved region. Several studies have shown that the conserved CX3C chemokine motif is associated with immune-mediated diseases following RSV infection [48–50], and anti-RSV G antibody responses can inhibit both CX3C in the RSV G-CX3CR1 interaction and RSV G-mediated leukocyte chemotaxis, consequently reducing disease pathogenesis [48, 51]. In our study, as mentioned above, a G/164-176-specific serum antibody was induced in both the GcfAB IN and SL groups, but the GcfAB IN group had a higher G/174-187-specific serum antibody titer than did the SL group. These antibody patterns may affect RSV G-CX3CR1-expressing cell interactions and CX3C motif-mediated leukocyte migration. As the GcfAB IN group showed less vaccine-mediated immunopathology than did the SL group, antibody reactivity against the B cell epitope of the RSV G protein may be an important factor in modulating RSV-associated pathogenesis.

Interestingly, GcfAB-immune mice inoculated via IN or SL routes generally showed lower levels of both eosinophilia and body weight loss following RSV B infection, indicating that GcfAB vaccination can protect against RSV B subtype without vaccine-induced immunopathology, in contrast with RSV A. Accordingly, G/183-195 of RSV B-specific CD4 T-cell responses were weak compared with those in the RSV A challenge groups. These results parallel those of a previous study showing that wtBGcf consisting of a.a. residues 131–230 of the G protein from the RSV B subtype did not elicit RSV B-specific CD4^+^ T-cell responses or pulmonary eosinophilia following RSV B challenge [29]. Perhaps a.a. 183–195 of the RSV B G protein might be a relatively weak CD4 T-cell epitope to process G-specific CD4^+^ T-cell responses, unlike the RSV A G protein. For other reasons, RSV A subtype is known to have a tendency to severe RSV-associated diseases compared to RSV B subtype [52, 53], or RSV B subtype used for our study, may not have fully adapted to the mouse. These factors might be associated with the differences between RSV A and B-associated immune responses and diseases.

In summary, our results demonstrate that mucosal immunization through IN or SL routes with GcfAB can induce strong humoral immunity that protects against both RSV subtype infections. Notably, SL administration with GcfAB plus CT elicited stronger Th17 responses and more severe vaccine-mediated pathology than did IN administration following RSV A infection, but not RSV B infection. Taken together, our results suggest that GcfAB could be used as a mucosal vaccine candidate preventing both RSV subtype infections, but caution is necessary with certain mucosal routes of administration.
